# Top-Down Control of Cortical State

**DOI:** 10.1016/j.neuron.2013.07.034

**Published:** 2013-08-07

**Authors:** Kenneth D. Harris

**Affiliations:** 1 UCL Institute of Neurology and UCL Department of Neuroscience, Physiology, and Pharmacology, 21 University Street, London WC1E 6DE, UK

## Abstract

Sensory cortices receive inputs not only from thalamus but also from higher-order cortical regions. Here, Zagha et al. (2013) show that motor cortical inputs can switch barrel cortex into a desynchronized state that enables more faithful representation of subtle sensory stimuli.

## Main Text

An animal’s reaction to a sensory stimulus depends on the context in which it is presented. In the cortex, even primary sensory areas receive a large number of “top-down” inputs from higher-order regions, in addition to the thalamic input that directly conveys sensory messages. These top-down connections are believed to underlie the integration of sensory inputs with nonsensory context.

One case in which a role for top-down cortical connections has been established is attention in the primate visual system. Strong electrical stimulation of the frontal eye fields (FEFs) produces eye movements to a topographically aligned location in space. However, weaker electrical stimulation—below the threshold for eliciting an overt saccade—instead mimics the effects of attention to this location, causing increased behavioral and neuronal responses to stimuli presented there (Moore and Armstrong, 2003).

In rodents, a robust experimental model of attention has not yet been established. However, there are remarkable parallels between the effects of attention on cortical processing in primates and changes in *cortical state* that occur with changes in behavioral context in rodents (Harris and Thiele, 2011). Cortical states were first described in relation to the sleep cycle. During slow-wave sleep, animals exhibit a *synchronized state*, characterized by large, slow fluctuations in the spiking and membrane potentials of large neuronal populations. By contrast, the cortex of awake, active, and alert animals exhibits a *desynchronized state* (also termed *activated state*) in which slow fluctuations are replaced by tonic cortical firing, often together with a higher-frequency gamma oscillation. Recent work has shown that these classical states are in fact points on a continuum. For example, quietly resting rodents show a moderately synchronized state, with fluctuations in cortical activity that are shallower and faster than classical sleep oscillations. When animals engage in active behaviors such as whisking or running, however, these moderate fluctuations are further reduced (Polack et al., 2013, Poulet et al., 2012).

There are several parallels between the correlates of selective attention in primates and cortical states in rodents. Their effects on local field potential oscillations are similar: when animals pay attention to a particular location in space, low-frequency oscillations are reduced in the aligned region of area V4, while high-frequency LFPs are increased (Fries et al., 2001). Attention and desynchronization both produce a decrease in trial-to-trial variability and noise correlation of sensory responses (Cohen and Maunsell, 2011, Goard and Dan, 2009, Marguet and Harris, 2011, Mitchell et al., 2009). Importantly, these effects only occur when attention is directed into the receptive fields of recorded neurons. This suggests that attention causes an effect similar to desynchronization, occurring locally in the region of visual cortex topographically aligned with the attended location. Moreover, recent results suggest that when attention is directed not to a region of space but to a visual feature, variability and correlation decrease in the population that encodes this feature (Cohen and Maunsell, 2011), suggesting that a phenomenon analogous to desynchronization has occurred in a spatially distributed neuronal assembly.

The mechanisms of cortical state change have been a subject of investigation for many decades. Classical research yielded two schools of thought on this question. The first, espoused by Steriade and colleagues, held that cortical states are modulated primarily via the thalamus. In this view, increased cholinergic input to thalamic relay cells leads to increased tonic firing and thus to a steady glutamatergic drive to cortex that causes desynchronization. The second perspective, espoused by Vanderwolf and colleagues, held that cortical state reflected direct neuromodulation of neocortex. Recent research provides support for both mechanisms. In the rodent somatosensory system, whisking causes increased tonic firing in thalamus; blocking thalamic firing with muscimol reduces the cortical depolarization caused by whisking, whereas stimulating thalamus optogenetically causes cortical desynchronization (Poulet et al., 2012). Support for direct cortical neuromodulation comes from the ability of locally applied neuromodulatory blockers to reduce the desynchronization caused by electrical stimulation of nucleus basalis or locomotion (Goard and Dan, 2009, Polack et al., 2013).

If attention does indeed consist of cortical state change occurring at a local level, one might expect the two phenomena to have similar circuit mechanisms. In particular, given the role of top-down cortical connections in attention, it has been hypothesized that tonic glutamatergic input from higher-order cortex should also cause desynchronization in rodent cortex (Harris and Thiele, 2011). The study of Zagha et al. (2013) provides direct evidence for this hypothesis.

Zagha et al. (2013) performed a number of elegant experiments to study the role of top-down connections from vibrissa motor cortex (vM1) to barrel cortex (S1). They found that blocking spiking in vM1 using muscimol shifted S1 toward more synchronized states, whereas optogenetically increasing vM1 activity shifted S1 toward more desynchronized states. This desynchronization was usually accompanied by an increase in firing rate of S1 neurons. Importantly, the effects on S1 state did not simply reflect the consequence of these manipulations on behavior. As might be expected, suppression or activation of vM1 activity caused a corresponding decrease or increase in the probability and amplitude of whisking. Nevertheless, an effect of manipulating vM1 on S1 state was seen even when analyzing data within whisking or nonwhisking periods. One can thus make an analogy between the effects of stimulating vM1 in rodents and the effects of stimulating FEF in primates: while strong enough stimulation of these areas causes an overt movement (saccade or whisking), weaker stimulation may instead produce covert effects on sensory processing in lower cortical areas (attention or desynchronization).

**Figure 1:**
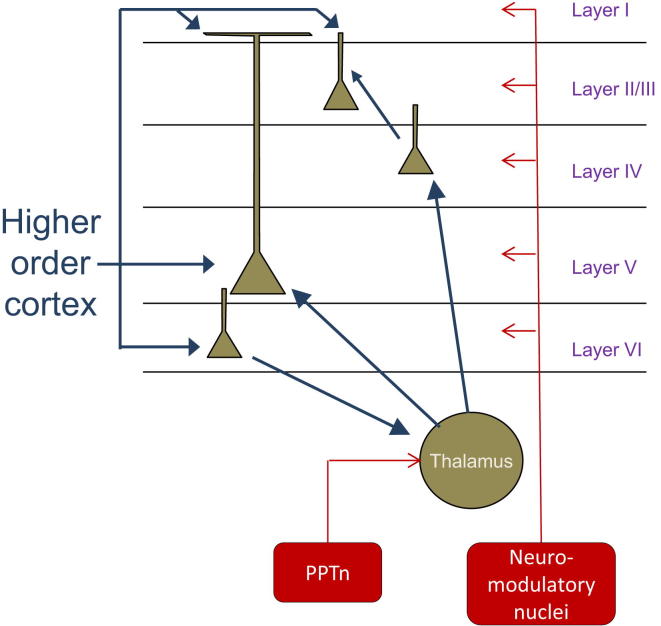
Three Mechanisms Contributing to Cortical Desynchronization First, as shown by Zagha et al. (2013), glutamatergic input from motor cortex can produce a desynchronized state in S1, together with an increased firing rate of S1 neurons. The increased rate of layer 2/3 neurons requires motor cortical axons in layer 1, whereas the increased rate of layer 5 neurons does not. Second, cholinergic input from the pendunculopontine tegmental nucleus (PPTn) to the thalamus increases tonic firing of thalamic relay cells, leading to cortical desynchronization. Corticothalamic inputs from layer 6 (itself a target of top-down feedback) might further contribute to thalamic activation. Third, direct control of cortical circuit neuromodulators such as acetylcholine and norepinephrine may further contribute to desynchronization. While all three mechanisms contribute to the decrease in low-frequency fluctuations that typify the desynchronized state, they may have different effects on other aspects of cortical processing, such as the firing rates of individual cell classes.

To investigate the mechanisms by which vM1 stimulation causes desynchronization of S1, Zagha et al. (2013) performed a series of further experiments. Current-source density analysis showed that vM1 stimulation produces sinks in layer 1 and layers 5/6, corresponding to the major termination zones of these cortical feedback axons. By applying varying concentrations of the glutamatergic antagonist CNQX, they showed that the increase in firing of superficial layer S1 neurons required layer 1 inputs, whereas inputs terminating in deep layers were sufficient for increased firing of layer 5 cells. To investigate whether stimulation of vM1 desynchronizes S1 via a direct pathway, without requiring additional relay stations, they performed additional tests. Optogenetic activation of vM1 could still desynchronize vS1 after suppressing activity in VPM thalamus; and optical stimulation of vM1 axons in S1 could still activate S1 even when the firing of vM1 somas was blocked to eliminate antidromic signaling. These data confirm that, in addition to the classical pathways that modulate cortical states, top-down projections are capable of directly desynchronizing sensory cortex (see Figure 1).

Cortical states have a complex effect on responses to sensory stimuli. Previous work has shown that the response to strong, sudden stimuli, such as tone onsets or whisker deflections is robust in both synchronized and desynchronized states (Castro-Alamancos, 2004, Luczak et al., 2013). However, more subtle, temporally extended stimuli such as natural movies, sustained tones, or repeated whisker deflections are represented more faithfully by the desynchronized cortex (Goard and Dan, 2009, Luczak et al., 2013, Marguet and Harris, 2011). Here one may again make an analogy with attention: strong, sudden stimuli which are capable of eliciting “bottom-up” attention are able to drive responses in either state, but faithful representation of weaker stimuli requires “top-down” attention in the form of cortical desynchronization. Zagha et al. (2013) investigated the effects of vM1-elicited desynchronization on the representation of a sequence of whisker deflections of random amplitudes. Consistent with this view, they found that the representation of low-amplitude whisker deflections was made more reliable by vM1 stimulation, but the representation of large-amplitude deflections was less affected.

This study has provided very important information on the function of top-down connections in rodent cortex, as well as further support for a close relationship between cortical state modulation and selective attention. However, the study also raises a number of further questions.

First, how specifically can top-down connections modulate sensory cortex in rodents? In primates, selective attention causes effects analogous to desynchronization in localized areas of cortex and even in anatomically distributed neuronal assemblies (Cohen and Maunsell, 2011, Fries et al., 2001, Mitchell et al., 2009). Stimulation of primate FEF causes increased sensory responses and reduced variability only in topographically aligned regions of V4 (Moore and Armstrong, 2003). How specific are the effects of top-down projections in rodent? Zagha et al. (2013) provide a partial answer to this question by showing that vM1 stimulation causes less desynchronization of visual cortex than of barrel cortex. But could projections from vM1 to vS1 selectively target a single whisker barrel or a distributed neuronal assembly? Recent experimental techniques involving retrograde viral gene delivery could potentially answer this question.

Second, how many dimensions has the space of cortical states? Zagha et al. (2013)’s study, together with previous work, shows that there are at least three circuit pathways that can contribute to cortical desynchronization: direct neuromodulation of cortex, increased tonic activity of thalamus, and increased corticocortical input (see Figure 1). Do these mechanisms produce identical effects, or are there subtle differences between them? There are reasons to suspect that the space of states is indeed multidimensional, i.e., that in addition to the common effect of reducing low-frequency fluctuations, different desynchronizing manipulations have diverse effects on cortical processing. For example, Zagha et al. (2013) showed that strong vM1 stimulation typically increases the firing rates of both of superficial and deep layer neurons. A similar effect was seen due to running in mouse V1 (Niell and Stryker, 2010), but desynchronizing brainstem stimulation (Sakata and Harris, 2012), or direct cholinergic manipulation of thalamus (Hirata and Castro-Alamancos, 2010), causes a desynchronization with suppressed superficial layer firing. Together with other examples (Harris and Thiele, 2011), these results suggest that the different pathways mediating cortical desynchronization have nonidentical effects on cortical processing. Given the number of ways that context can affect stimulus perception, one should expect the neural circuits producing nonsensory control of cortex to be highly complex. The study of Zagha et al. (2013) provides a very important step toward understanding this circuitry.
